# Validation of the Simplified Chinese Clear Communication Index Using Diabetes Education Materials: Instrument Adaptation and Validation Study

**DOI:** 10.2196/83935

**Published:** 2026-02-20

**Authors:** Lu Liu, Yi Chen, Xiaoqi Li, Hanyu Du, Rong Gui, Huiqing Jin, Fengfei Chen, Yujun Lin, Qiuhuan Huang, Yingge Tong

**Affiliations:** 1 Department of Nursing, School of Public Health and Nursing, Hangzhou Normal University, No. 2318, Yuhangtang Road, Hangzhou, 311121, China, 8615785423928; 2 Department of Biomedicine and Prevention, University of Rome Tor Vergata, Rome, Italy; 3 Affiliated Hospital of Youjiang Medical University for Nationalities, Baise, Guangxi Zhuang Autonomous Region, China; 4 Guangxi Clinical Medical Research Center for Hepatobiliary Diseases, Baise, Guangxi Zhuang Autonomous Region, China

**Keywords:** clear communication index, psychometric validation, cross-cultural adaptation, health literacy, health education materials, public health communication, diabetes education, simplified Chinese

## Abstract

**Background:**

In the postpandemic context, the surge of digital health information has intensified public demand for clear and practical communication, particularly in China, where health literacy disparities persist. The Clear Communication Index, developed by the US Centers for Disease Control and Prevention (CDC), is a standardized tool for assessing the clarity and actionability of health materials, but no version adapted to the simplified Chinese context has been established.

**Objective:**

This study aims to translate and culturally adapt the Clear Communication Index into simplified Chinese, and subsequently validate its psychometric properties using diabetes health communication materials from provincial CDCs across Mainland China, a disease area with a substantial public health burden and strong reliance on health education.

**Methods:**

Following a standardized cross-cultural process (forward-back translation and expert review), we developed the simplified Chinese version of the Clear Communication Index (C-CCI) and finalized a 12-item scale across 4 dimensions (Main Message and Call to Action, Behavioral Recommendations, Numbers, Risk) with yes or no scoring (0‐100). One top-ranked diabetes health education material was sampled from each provincial CDC website in Mainland China (30/31 included; 96.8%) on May 18, 2025. Twelve raters with multidisciplinary backgrounds completed a 3-week standardized training program and independently evaluated each article (360 ratings). Structural validity was examined using exploratory factor analysis and confirmatory factor analysis. Content validity was assessed by the item-level content validity index and the scale-level content validity index/average. Reliability was evaluated by internal consistency (Cronbach α) and inter-rater agreement (Fleiss kappa), while convergent/discriminant validity was assessed using composite reliability and average variance extracted (AVE).

**Results:**

The 4-factor structure was supported. Content validity was high, with the scale-level content validity index/average values of 0.976 for clarity and 1 for relevance. Overall reliability was acceptable (Cronbach α=0.837), with particularly strong internal consistency in the Risk dimension (Cronbach α=0.910). Inter-rater agreement was substantial (κ=0.624). Convergent validity (composite reliability=0.897‐0.914; AVE=0.645‐0.831) and discriminant validity were satisfactory. Application to 30 provincial CDC websites yielded a mean C-CCI score of 53.84 (SD 29.74), well below the recommended threshold of 90. No significant regional differences were observed in total scores; however, Behavioral Recommendations scored slightly higher in western provinces than in eastern and central regions (η²=0.034), representing a small effect size with limited practical significance.

**Conclusions:**

The C-CCI demonstrated good validity, reliability, and feasibility for evaluating simplified Chinese health communication materials. These findings underscore the need to strengthen health communication practices in China and encourage provincial CDCs to align material development with national health literacy goals. Integrating the C-CCI into routine CDC review protocols could support evidence-based quality assurance and advance clearer, more actionable public health communication nationwide.

## Introduction

Effective communication of health information is critical to facilitating accurate public understanding and proper application [1]. In the postpandemic era, both public attention to and demand for health information have increased significantly [2]. However, limited health literacy remains a widespread challenge worldwide, affecting about 88% of adults in the United States, nearly 70% in China, and up to 62.1% in parts of Europe [3-5]. Complex medical terminology and dense sentence structures often impede comprehension, especially among populations with limited literacy, contributing to poor adherence, adverse outcomes, and higher mortality [6,7]. These challenges highlight the need for clear, actionable, and audience-appropriate health materials.

To address these challenges, several standardized tools have been developed internationally to evaluate the quality of health communication materials, including the Suitability Assessment of Materials (SAM) [8], the Patient Education Materials Assessment Tool (PEMAT) [9], and the Clear Communication Index (CCI) [10]. These instruments differ in both scope and analytical emphasis. SAM adopts a broad suitability-oriented framework emphasizing presentation and cultural appropriateness, while PEMAT focuses on understandability and actionability. However, neither tool explicitly prioritizes key messages or systematically integrates numeric and risk communication.

In addition, several tools, including the Credible and Usable Evaluation of patient education tool for websites [11], the Health Information Website Evaluation Tool [12], the Health Information Quality Assessment Tool [13], and the QUality Evaluation Scoring Tool [14], have been developed to assess the quality and credibility of health information, particularly for professional or web-based contexts. However, their primary focus on credibility and usability limits their applicability to audience-centered, action-oriented evaluation of public health materials.

In contrast to these approaches, the CCI incorporates audience-centered principles, prioritizing key messages, numerical clarity, and behavioral guidance, making it a more comprehensive and action-oriented evaluation framework [10]. Among these instruments, the SAM and PEMAT have been translated and validated in simplified Chinese [15,16]; however, a validated simplified Chinese version of the Clear Communication Index (C-CCI) has not yet been developed.

Developed by the US Centers for Disease Control and Prevention (CDC) in 2014, the CCI provides a practical and evidence-based method for assessing the clarity of health materials [10]. It is available in a full version (20 items across 7 dimensions) for longer materials and a modified version (13 items across 4 dimensions) designed for short-format communications, both using binary scoring with a recommended minimum score of 90 to indicate acceptable clarity and usability [10,17,18]. The CCI has been widely applied across health domains, including patient education, cancer communication, and environmental health [19-27], and cross-culturally validated in countries, such as Brazil and Thailand [28,29]. A recent systematic review classified the CCI as a Grade B recommended instrument for health communication evaluation [30].

However, no validated simplified C-CCI currently exists. Given linguistic and cultural differences between Chinese and English, direct adoption may limit its accuracy and relevance [31]. In China, health communication is largely delivered through a nationwide network of CDCs that translate national health policies into public education, particularly for chronic disease prevention and management [32]. Diabetes, as a major noncommunicable disease, represents a central focus of CDC health education across provinces. Given provincial CDCs’ mandate to bridge policy objectives and public engagement across regions, a standardized, context-sensitive framework for evaluating their educational materials is needed.

To address this gap, this study aimed to translate and culturally adapt the original CCI into a simplified Chinese version (C-CCI) through a standardized cross-cultural process and to evaluate its reliability and validity using health communication materials from provincial CDCs across Mainland China.

## Methods

### Translation and Cultural Adaptation of the CCI Tool

**Figure 1.**
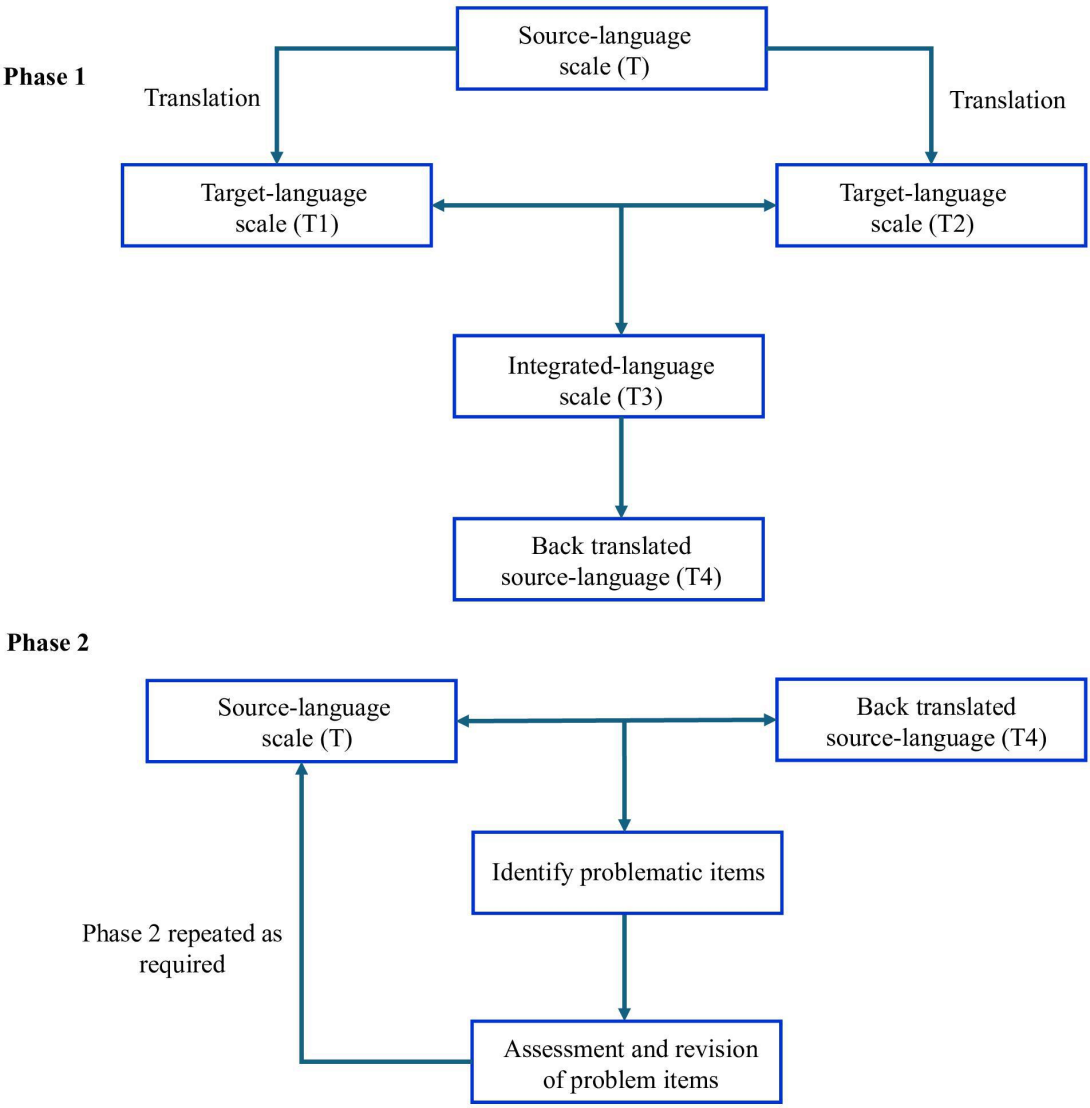
Process of developing the modified simplified Chinese version of Clear Communication Index.

After obtaining formal authorization to use and adapt the original CCI, the research team followed the standardized cross-cultural adaptation model proposed by Sperber et al [33]. The process was conducted in 4 stages. First, 2 native simplified Chinese translators with expertise in health communication independently produced forward translations of the instrument (T1 and T2). Then, these were compared and reconciled by a senior bilingual translator with a medical background, who synthesized them into a single version (T3). To verify the linguistic equivalence, T3 was subsequently back-translated into English (T4) by an independent professional bilingual translator who had no prior knowledge of the tool and was not involved in the forward translation process, thereby minimizing potential bias and ensuring semantic fidelity. Finally, to further evaluate translation quality, we applied the linguistic equivalence assessment method recommended by Sperber et al [33]. This method involved comparing the original English version with the back-translated version across 2 dimensions. The first dimension is comparability of language, which refers to the similarity of words, phrases, and sentence structures. The second dimension is similarity of interpretability, which refers to the extent to which different wordings convey equivalent meaning. A linguist with a doctoral degree conducted this assessment using 4-point ordinal scales (1=extremely comparable or similar; 2=comparable or similar; 3=not comparable or similar; 4=not at all comparable or similar). Items rated as 3 or 4 on either dimension were reviewed by an expert panel consisting of one health education specialist and 2 nurses**,** all of whom had formal training in health communication and substantial experience in patient-facing health education and counseling. Revisions were made through iterative panel discussions until consensus was reached. When the initial agreement was unattainable, additional rounds of discussion were used to determine the most contextually and semantically appropriate wording. The finalized items were then incorporated into the final C-CCI. The overall process is summarized in Figure 1.

### Reliability and Validity Evaluation of the C-CCI

#### Content Validity

Following Polit and Beck’s guidelines for establishing content validity [34], 7 experts were recruited to ensure methodological rigor and representativeness. The expert panel comprised 7 raters (2 male and 5 female), with a mean age of 41 years (SD 5.8). All experts held senior professional titles. Their backgrounds in health education, chronic disease nursing, and nursing education provided a balance of clinical and educational perspectives for evaluating the C-CCI items. The evaluation focused on 2 dimensions: language clarity and content relevance. Experts independently rated each item using a 4-point Likert scale. For clarity, scores ranged from 1=unclear to 4=clear; for relevance, from 1=irrelevant to 4=relevant. Based on these ratings, both the item-level content validity index (I-CVI) and the scale-level content validity index/average (S-CVI/Ave) were calculated. The I-CVI was computed as the proportion of experts rating an item as 3 or 4. The S-CVI/Ave was calculated as the average item-level CVIs, reflecting the overall content validity of the instrument. According to published recommendations, an S-CVI/Ave above 0.90 is considered acceptable; for panels of ≤5 experts, an I-CVI of 1 is required, whereas for panels of >5 experts, the minimum acceptable I-CVI is 0.78 [34,35].

#### Exploratory Factor Analysis

An exploratory factor analysis (EFA) was conducted to investigate the latent structure of the C-CCI scale. Prior to factor extraction, the Kaiser–Meyer–Olkin (KMO) test and Bartlett test of sphericity were performed to evaluate the suitability of the data. When the KMO value exceeded 0.60 and the Bartlett test yielded statistically significant results (*P*<.05), the data were deemed suitable for factor analysis [36]. Factors were extracted using principal component analysis. As prespecified in the analysis plan, the choice of rotation method was determined by the inter-factor correlations: when correlation coefficients exceeded 0.30, oblique rotation was applied; otherwise, orthogonal rotation was used [37]. Items were retained according to the following criteria: factor loading below 0.40, cross-loadings on multiple factors with a difference of less than 0.25. Items meeting any of these conditions were flagged for potential deletion [38].

#### Confirmatory Factor Analysis

Based on the factor structure derived from the EFA, a confirmatory factor analysis (CFA) was conducted to assess model fit. The assessment was performed at 2 levels: preliminary fit and overall fit indices. Preliminary criteria required all error variances to be positive, t-values of parameter estimates to reach statistical significance (*P*<.05), standard errors to be small, and factor loadings to fall within the range of 0.50‐0.95.

Overall model fit was primarily evaluated using the root mean square error of approximation (RMSEA) and the comparative fit index (CFI), which are widely recommended as core indices for assessing model adequacy [39,40]. RMSEA values below 0.08 were considered indicative of acceptable fit, and values of CFI≥0.90 reflected good model fit. As complementary indices, the standardized root mean square residual (<0.08), the incremental fit index and Tucker–Lewis index (≥0.90), as well as the goodness-of-fit index (≥0.90), were also examined to provide a more comprehensive evaluation of model performance [39-41]. In addition, chi-square to degrees of freedom ratio (*χ*²/*df*)<3 was considered indicative of good fit, whereas values between 3 and 5 were regarded as acceptable [36].

### Convergent and Discriminant Validity

Composite reliability (CR) and average variance extracted (AVE) were calculated to evaluate convergent validity [42]. Convergent validity was considered strong when CR exceeded 0.70 and AVE was greater than 0.50, and acceptable when AVE ranged between 0.36 and 0.50 [43]. Discriminant validity was assessed by comparing the square root of the AVE for each construct with the inter-construct correlations. When the square root of AVE for a construct was greater than its correlations with other constructs, discriminant validity was deemed satisfactory [42].

### Reliability

To assess the reliability of the C-CCI, Fleiss kappa (κ) test was applied to evaluate inter-rater agreement, and Cronbach α coefficient was computed to examine internal consistency. A κ value between 0.61 and 0.80 was interpreted as indicating substantial agreement [44], whereas a Cronbach α coefficient greater than 0.70 was deemed indicative of acceptable internal consistency [45].

### Data Collection

A comprehensive search of the official websites of all 31 provincial CDCs in Mainland China was conducted, focusing on diabetes as the evaluation theme given its global rise, major public health burden, and reliance on health education for prevention and management [46,47]. The search was completed on May 18, 2025.

The keyword “diabetes” was used in the internal search engines of the provincial CDC websites to ensure accuracy and consistency. Consistent with the “least effort” principle, whereby the public typically focuses on top-ranked results [48], the highest-ranked diabetes-related health article from each provincial CDC was selected for analysis. Exclusion criteria included government reports, bidding information, and policy or regulatory documents exceeding 1000 words. The Uniform Resource Locators of the selected articles were compiled into an Excel database for systematic evaluation. The Jilin Provincial CDC website did not provide relevant content and was therefore excluded. In total, 30 provincial-level CDC websites were included, covering regions with diverse economic development levels and cultural backgrounds, thereby ensuring a nationally representative sample.

Nine graduate nursing students and 3 senior nurses with more than 5 years of endocrinology nursing experience were invited as evaluators. Prior to the formal evaluation, all evaluators completed a 3-week systematic training that covered the theoretical foundation of the tool, scoring criteria, and application methods. Training included multiple discussions and simulated scoring sessions to enhance the consistency and understanding of the tool’s dimensions. After the training, twelve raters independently evaluated health education materials collected from 30 provinces, producing a total of 360 data entries. To ensure stable and reliable results, factor analysis requires an adequate sample size. Recommendations vary, with common rules of thumb suggesting a minimum of 100 to 200 participants, or a subject-to-item ratio of at least 5:1 or 10:1 [49]. CFA, in particular, requires larger samples to achieve stable parameter estimates and sufficient statistical power [50]. Simulation-based studies using Monte Carlo methods indicate that sample size requirements for CFA depend more on model complexity, factor loadings, and indicator structure than on fixed numerical thresholds [50]. Under typical psychometric conditions, about 200 participants generally provide sufficient power for models of moderate complexity [50]. Accordingly, this study used a 2-step validation process using 2 independent samples: used 2 independent samples: 150 cases for EFA and 210 for CFA, the latter yielding more than 0.80 power for detecting RMSEA deviations at 0.08 [50], ensuring model stability and reliable fit evaluation.

### Data Analysis

All statistical analyses were conducted using IBM SPSS Statistics (version 27.0; IBM Corp) and IBM SPSS Amos (version 28.0; IBM Corp). Based on the study design, consistency tests, reliability analyses, EFA, and CFA were performed to assess the scale’s reliability and validity. All analyses were 2-tailed, and a *P* value <.05 was considered statistically significant.

### Ethical Considerations

This study was approved by the Ethics Committee of Hangzhou Normal University (Approval No. 2025063). All participants provided informed consent before participating. All rater data were anonymized and deidentified prior to analysis, and the evaluated health education materials were obtained from publicly accessible sources. All evaluation data were coded, securely stored, and used solely for research purposes. The study was conducted in accordance with relevant institutional guidelines and the Declaration of Helsinki. All participants involved in this study were provided a small token of appreciation in the form of a gift worth approximately 30 RMB (US $4.13).

## Results

### Comparison Between Original and Back-Translation, and Cultural Adaptation

Comparison of the original and back-translated versions showed strong conceptual and semantic equivalence (Table 1). Across all items, the mean score for comparability of language was 1.308 (SD 0.63), and the mean score for SI was 1.077 (SD 0.28), indicating a high level of linguistic and interpretive consistency between the 2 versions. Overall variation across items was minimal, with the exception of item 3. While the phrase “policy change” can be directly translated, the research team cautioned that, within the context of public health in Mainland China, it may be misconstrued as implying radical “reform.” To avoid this potential misinterpretation, the phrase was revised to “alignment with national health policies,” thereby softening the connotation of drastic change and emphasizing policy continuity and optimization.

**Table 1.** This section compares the original and back-translated English versions of the Clear Communication Index Score sheet in terms of language comparability and interpretability similarity.

Original English version	Back-translated English version	Comparability of language	Similarity of interpretability
Does the material contain one main message statement? A main message is the one thing you want to communicate to a person or group that they must remember.	Does the material have a core message? The core message is what you want to convey to individuals or groups and wish them to remember.	2	1
Is the main message at the top, beginning, or front of the material? The main message must be in the first paragraph or section. A section is a block of text between headings.	Is the core message at the top, beginning, or front of the material? It must appear in the first paragraph or section. A paragraph is a block of text between headings.	1	1
Does the material include one or more calls to action for the primary audience? If the material includes a specific behavioral recommendation, a prompt to get more information, a request to share information with someone else, or a broad call for program or policy change, answer yes.	Does the material contain one or more calls to action for the main audience? If the material includes any of the following—for example, specific behavioral recommendations, guidance on how to access authoritative health information, encouragement to share the information with others, or a call to action aligned with national health policy directives.	3	1
Do both the main message and the call to action use the active voice?	Are both the core message and call to action in active voice?	1	1
Does the material always use words the primary audience uses?	Is the wording always appropriate to the main audience's habits?	2	2
Is the most important information the primary audience needs summarized in the first paragraph or section?	Is the most important information for the target audience outlined in the first paragraph or section?	1	1
Does the material include one or more behavioral recommendations for the primary audience?	Does the material offer one or more behavior recommendations for the target audience?	1	1
Does the material explain why the behavioral recommendation(s) is important to the primary audience?	Does it explain the importance of the recommended actions to the main audience?	1	1
Does the material always present numbers the primary audience uses?	Are numbers in the material always presented in a way that's familiar to the target audience?	1	1
Does the audience have to conduct mathematical calculations?	Does the audience need to do math?	1	1
Does the material explain the nature of the risk?	Does the material explain the nature of the risk?	1	1
Does the material address both the risks and benefits of the recommended behaviors? This includes actual risks and benefits and those perceived by your audience.	Does it describe the precautions and benefits of the recommended actions? This includes actual and perceived risks and benefits.	1	1
If the material uses numeric probability to describe risk, is the probability also explained with words or a visual?	If the material uses numbers (eg, 1/5 or 20%) to describe risks and also explains these probabilities with words or illustrations, select "yes".	1	1

To further enhance comprehensibility and applicability in the Mainland simplified Chinese context, culturally specific expressions were localized while maintaining consistency with the original scoring framework. For example, in the “NOTE” section of the scale, “Facebook posts,” “Twitter messages,” “scripts for podcasts” and “call center responses” were replaced with “WeChat official account articles,” “Weibo posts” “short video scripts” and “customer service responses” respectively. These adaptations enhanced cultural relevance and user understanding.

### Validity and Reliability of the C-CCI

#### Content Validity

The scale demonstrated high content validity, with overall CVIs of 0.976 for clarity and 0.934 for relevance. Item-level CVIs ranged from 0.857 to 1 for clarity and from 0.124 to 1 for relevance. The notably low relevance score was attributable to Item 4 under the dimension “Main Message and Call to Action,” which focused on the use of passive voice. Passive constructions, though acceptable in English health communication, are uncommon in simplified Chinese and may reduce clarity and perceived relevance. This reflects a cross-linguistic mismatch between formal and functional equivalence, consistent with Nida’s theory of dynamic equivalence. Accordingly, Item 4 was deemed culturally unsuitable and removed [51]. After its removal, all items achieved a relevance index of 1, yielding a final scale of 12 items (Table 2).

**Table 2.** The reliability and content validity of the simplified Chinese version of the Clear Communication Index.

Variables	Cronbach α (N=360)	I-CVI^a^ (n=7)	S-CVI^b^ (n=7)
Main message and call to action	0.861	—^c^	—
Behavioral recommendations	0.894	—	—
Numbers	0.827	—	—
Risk	0.910	—	—
Total	0.837	0.857~1.000^d^ or 1^e^	0.976^d^ or 1^e^

a I-CVI: item-level content validity index.

b S-CVI: scale-level content validity index.

c Not applicable.

d For language clarity.

e For content relevance.

### EFA

**Figure 2.**
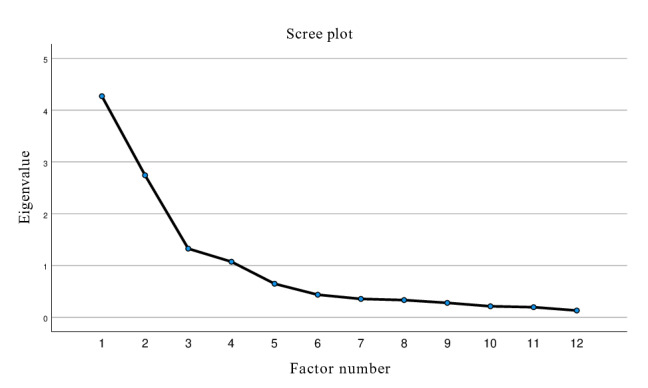
Scree plot of Eigenvalues for exploratory factor analysis of the simplified Chinese version of the Clear Communication Index.

The KMO value was 0.769, and Bartlett test of sphericity was significant (*χ*²_66_=1034.141; *P*<.001), indicating the data’s suitability for factor analysis. Four factors with eigenvalues greater than 1 were extracted, accounting for 78.7% of the total variance, consistent with the factor structure of the original scale. The scree plot also supported a 4-factor solution, with a clear inflection after the fourth factor (Figure 2).

Item loadings (Table 3) were generally high and well defined, and all items showed satisfactory communalities, ranging from 0.475 to 0.903 [52]. Although item 4 had a slightly lower loading (0.484), it exceeded the 0.40 retention threshold and demonstrated adequate communality and semantic alignment within the “Main Message and Call to Action” dimension; thus, it was retained. Overall, the EFA indicated a clear and stable factor structure with well-defined dimensions, providing strong support for the construct validity of the C-CCI.

**Table 3.** Factor loadings of exploratory factor analysis for the simplified Chinese version of the Clear Communication Index.

Items	Factor 1	Factor 2	Factor 3	Factor 4	Communality
A1	0.810	—^a^	—	—	0.711
A2	0.835	—	—	—	0.705
A3	0.863	—	—	—	0.769
A4	0.484	—	0.435	—	0.475
A5	0.878	—	—	—	0.810
B6	—	0.905	—	—	0.888
B7	—	0.922	—	—	0.903
C8	—	—	0.862	—	0.789
C9	—	—	0.890	—	0.830
D10	—	—	—	0.913	0.899
D11	—	—	—	0.819	0.785
D12	—	—	—	0.911	0.847

a Loadings < 0.40 were suppressed.

### CFA

**Figure 3.**
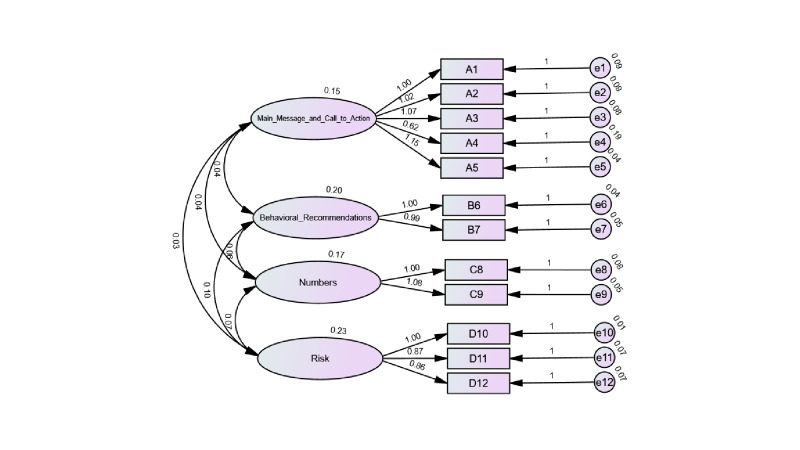
Standardized 4-factor model of the simplified Chinese version of Clear Communication Index.

As shown in Figure 3, the CFA results supported the 4-factor structure of the C-CCI. Model fit indices indicated an excellent fit: *χ²_48_=90.95 (χ*²/*df*=1.895), suggesting strong model adequacy; RMSEA=0.065, further supporting the good fit assumption. The CFI value was 0.972, exceeding the conventional cutoff of 0.90 and indicating strong model fit. Other indices also performed well: goodness-of-fit index=0.934, incremental fit index=0.972, Tucker–Lewis index=0.961, and standardized root mean square residual=0.072 (Table 4). All standardized factor loadings were substantial, ranging from 0.70 to 0.95, with each indicator loading strongly on its intended latent construct. No post hoc model respecifications were made based on modification indices, and the final model was retained as theoretically specified. These results demonstrate an excellent overall model fit, with stable and theoretically coherent parameter estimates across the 4-factor structure.

**Table 4.** Model fit index for the simplified Chinese version of the Clear Communication Index.

Index	Four-factor	Evaluation standard
*χ*² (*df*)	90.95 (48)	≤3
GFI^a^	0.934	>0.900
CFI^b^	0.972	>0.900
IFI^c^	0.972	>0.900
TLI^d^	0.961	>0.900
RMSEA^e^	0.065	<0.080
SRMR^f^	0.072	<0.080

a GFI: goodness-of-fit index.

b CFI: comparative fit index.

c IFI: incremental fit index.

d TLI: Tucker–Lewis index.

e RMSEA: root mean square error of approximation.

f SRMR: standardized root mean square residual.

### Convergent and Discriminant Validity

CR values were 0.907, 0.897, 0.914, and 0.897 for Main Message and Call to Action, Behavioral Recommendations, Numbers, and Risk, respectively. The AVE values were 0.831, 0.814, 0.774, and 0.645, demonstrating strong convergent validity. Discriminant validity was assessed using the Fornell–Larcker criterion [43], comparing the square root of each construct’s AVE with its inter-construct correlations (Table 5). In all cases, the square root of AVE exceeded its highest correlation with any other factor. Specifically, the Risk construct exhibited a square root of AVE of 0.803, showed a higher value than its strongest correlation with Behavioral Recommendations (*r*=0.442), and the same pattern was observed for Main Message and Call to Action (0.911), Behavioral Recommendations (0.902), and Numbers (0.880). Although moderate correlations were observed among some constructs, these findings indicate that the 4 dimensions are empirically distinct while remaining theoretically related.

**Table 5.** Convergent validity and discriminant validity of the simplified Chinese version of the Clear Communication Index.

Convergent validity and discriminant validity	Risk	Numbers	Behavioral recommendations	Main message and call to action	AVE^a^	CR^b^
Risk	0.803	—	—	—	0.645	0.897
Numbers	0.371^c^	0.880	—	—	0.774	0.914
Behavioral recommendations	0.442^c^	0.320^c^	0.902	—	0.814	0.897
Main message and call to action	0.152^c^	0.221^c^	0.241^c^	0.911	0.831	0.907

a AVE: average variance extracted.

b CR: composite reliability.

c *P*<.01.

### Reliability

Expert ratings demonstrated substantial consistency (κ=0.624). The scale demonstrated high internal consistency overall (Cronbach α=0.837), with dimension-specific coefficients of 0.861 (Main Message and Call to Action), 0.894 (Behavioral Recommendations), 0.827 (Numbers), and 0.910 (Risk), confirming strong reliability across dimensions.

### Evaluation of Diabetes Health Communication by Provincial CDCs in Mainland China

Of the 31 provincial-level administrative divisions in Mainland China, 30 CDC websites were included (with a coverage of 96.8%), ensuring strong national representativeness. Among diabetes-related health education materials, one province (Qinghai) provided video content with text captions, 8 used text–image formats, and 21 (70%) provided text-only content. Provinces were categorized into eastern, central, and western economic regions in Mainland China [53]. Mean C-CCI scores did not differ significantly across regions (East: mean 50.06, SD 29.06; Central: mean 55.26, SD 35.52; and West: mean 56.48, SD 26.32). The national mean score was 53.84 (SD 29.74), which fell well below the recommended threshold of 90, indicating considerable room for improvement in health communication quality. No regional differences were found across dimensions except for Behavioral Recommendations, where scores in the western region were significantly higher than those in the eastern and central regions (*P*=.002). However, the effect size was small (η²=0.034), suggesting that regional grouping accounted for only a minor proportion of the variance in Behavioral Recommendations (Table 6).

**Table 6.** Comparison of scores across dimensions in Eastern, Central, and Western Regions of Mainland China (N=360).

Dimensions and regions	n (%)	Mean (SD)	*F* test (*df*)	*P* value	LSD^a^
Main message and call to Action			0.152（2）	.86	—^b^
East	132 (36.7)	2.7 (1.91)			
Central	84 (23.3)	2.83 (2.21)			
West	144 (40)	2.82 (1.94)			
Overall	360 (100)	2.78 (1.99)			
Behavioral recommendations^c^			6.271（2）	.002	East＜West, *P*=.001; Central＜West, *P*=.008
East	132 (36.7)	0.98 (0.95)			
Central	84 (23.3)	1 (0.96)			
West	144 (40)	1.34 (0.89)			
Overall	360 (100)	1.13 (0.94)			
Numbers			1.399 (2)	.25	—
East	132 (36.7)	1.09 (0.89)			
Central	84 (23.3)	1.3 (0.86)			
West	144 (40)	1.13 (0.96)			
Overall	360 (100)	1.16 (0.91)			
Risk			1.457 (2)	.23	—
East	132 (36.7)	1.23 (1.46)			
Central	84 (23.3)	1.5 (1.41)			
West	144 (40)	1.49 (1.27)			
Overall	360 (100)	1.4 (1.38)			
Total score			1.735 (2)	.18	—
East	132 (36.7)	50.06 (29.06)			
Central	84 (23.3)	55.26 (35.52)			
West	144 (40)	56.48 (26.32)			
Overall	360 (100)	53.84 (29.74)			

a LSD: least significant difference.

b Not significant (*P *≥ .05)

c η² represents the effect size, indicating the proportion of total variance explained by regional grouping. For the Behavioral Recommendations dimension, η² = 0.034, indicating a small effect size.

## Discussion

### Reliability and Validity of the C-CCI Under a Rigorous Sinicization Process

To our knowledge, this study represents the first rigorous and systematic cross-cultural adaptation process to translate, culturally adapt, and psychometrically validate the CCI, resulting in the C-CCI. Raters with substantial professional experience underwent structured training encompassing theoretical foundations, scoring standards, and simulated evaluations. This process enhanced scoring consistency and minimized subjective bias, strengthening the methodological rigor of data collection. The C-CCI retained the 4 original dimensions: Main Message and Call to Action, Behavioral Recommendations, Numbers, and Risk, thereby preserving theoretical coherence, a structure that was further supported by EFA and confirmed through CFA. Targeted revisions improved cultural applicability, including revising item 3 from “policy change” to “alignment with national health policies” and deleting item 4 due to the incompatibility of passive voice in simplified Chinese. The deletion of items does not alter the scoring rules and, consequently, has no impact on the scoring threshold of the C-CCI. Following these adjustments, expert content validity scores improved, confirming the scientific soundness of the modifications. Psychometrically, the C-CCI demonstrated robust validity and reliability that not only met but exceeded international benchmarks for comparable Health Information Quality Assessment Tools. Prior reviews have shown that most instruments remain at Grade B due to limited structural validation and modest explained variance [30]. In contrast, the C-CCI achieved a cumulative variance of 78.7% in EFA and excellent model fit in CFA (RMSEA=0.065 and CFI=0.972), exceeding Grade B thresholds and reflecting superior structural coherence. Convergent validity was further supported by high CR and AVE values, while strong internal consistency (Cronbach α>.80) and substantial interrater agreement (κ=0.624) confirmed the instrument’s stability and reproducibility.

### Applicability and Promotion Potential of the C-CCI

Using diabetes health communication materials from 30 provincial CDC websites in Mainland China, including text-only, text-image, and video formats, this study systematically evaluated the real-world applicability of the C-CCI. The tool showed a clear structure and specific scoring dimensions, and raters achieved strong inter-rater reliability after brief training, demonstrating strong practicality and replicability in applied settings. The C-CCI effectively identified weaknesses in health communication materials across 4 dimensions, demonstrating strong problem detection capacity and intervention guidance value. Its stable performance across different regions and formats (text only, text-image, and video) further supports its adaptability to diverse contexts.

Importantly, the C-CCI aligns well with China’s evolving digital communication ecosystem, in which health information is increasingly disseminated through official WeChat (Tencent) accounts, TikTok (ByteDance) videos, and other interactive platforms. Although these channels differ in format and engagement mechanisms, they share core demands for message clarity, concise information hierarchy, and actionable behavioral guidance, principles directly reflected in the C-CCI framework. From an implementation perspective, provincial CDCs may use the tool as a formative evaluation instrument during content development and internal review, using it to guide message structuring and action-oriented framing prior to dissemination. This approach enables consistent quality assurance across regions while maintaining flexibility in adapting to platform-specific communication styles.

Beyond the Mainland China context, the C-CCI’s methodological foundation may also hold relevance for other Chinese-speaking regions that share similar linguistic and readability challenges, mirroring Mainland China communication patterns noted in previous studies [54-58]. However, its broader application should be guided by further adaptation studies that consider local policy environments and platform ecosystems, ensuring evidence-based contextualization rather than direct transfer.

### Comparison With International Adaptations and Added Value of This Study

The CCI has been adapted into multiple languages and contexts worldwide. For instance, the Brazilian Portuguese version demonstrated applicability among primary health care professionals through field validation [28], while the Thai version emphasized dissemination of the theoretical framework and facilitated adoption in local health education [29]. These studies collectively established the feasibility of cross-cultural adaptation but were limited in the scope of psychometric testing and in the range of evaluated materials. Compared with prior validations, this study advances the methodological frontier of CCI research through multiformat empirical testing across 30 provincial CDC websites. By systematically evaluating diabetes-related materials in text-only, text-image, and video formats, the study assessed whether the measurement properties of the C-CCI remained stable across media that differ substantially in information organization. This format inclusivity, supported by consistent reliability across modalities, broadens the empirical use of the CCI framework beyond single-medium applications.

In addition, the study incorporated a more comprehensive psychometric evaluation strategy, combining exploratory and confirmatory factor analyses with rigorous validity and reliability testing. The convergence of large-scale institutional sampling and cross-format validation provides a robust methodological blueprint for future adaptations in diverse health communication settings. This study also contributes a transferable model for standardizing and assessing health information quality across varied media environments.

### Quality of Diabetes Health Communication in Mainland China and Improvement Strategies

Using the C-CCI, this study systematically assessed the quality of diabetes health communication in mainland China, identifying overall quality levels, regional variation, and areas for improvement. Median scores across the 4 dimensions were approximately half of the maximum, revealing substantial room for enhancement. The national mean score of 53.84 (SD 29.74) fell below the recommended threshold of 90 [10], underscoring deficiencies in information completeness, structural coherence, and balance across dimensions.

Regional comparisons showed that western provinces scored significantly higher in Behavioral Recommendations than eastern and central provinces. However, the magnitude of this difference was small (η²=0.034), indicating that regional grouping explained only a limited proportion of the variance in Behavioral Recommendations. This pattern may be partly influenced by unmeasured contextual factors (eg, regional economic capacity, resource distribution, and health communication infrastructure), pending future covariate adjustment and mediation analyses. Given the heterogeneous developmental landscape across and within China’s provinces, aggregation into broad east–central–west categories may obscure underlying structural disparities and inflate within-group variance. For example, within the eastern region, provinces, such as Hainan and Hebei, may exhibit comparatively lower development profiles than other eastern provinces, while within the western region, provinces and municipalities, such as Sichuan and Chongqing, may demonstrate development levels comparable to, or even exceeding, those of some central or eastern provinces. Similar internal disparities may also be present within the central region. These findings underscore the importance of interpreting statistically significant regional differences alongside effect size estimates. Hence, future research should adopt more fine-grained, mechanism-sensitive grouping strategies or multilevel analytical frameworks, incorporating appropriate covariate adjustment and mediation analyses to distinguish structural influences from context-specific practices. Based on standardized percentage scores, Risk emerged as the lowest-performing dimension, followed by Main Message and Call to Action, with Behavioral Recommendations and Numbers showing relatively better performance. Accordingly, improvement efforts should be prioritized in alignment with these empirical deficits: (1) enriching risk communication should be the foremost priority. This could involve contextualizing both absolute and relative risk information in line with evidence-based communication principles, supported by standardized templates and iterative user testing; (2) strengthening the clarity and authority of main messages should follow, through improved headline structuring, message salience testing (eg, digital A/B testing), and inclusion of credible source attribution; (3) improving numerical presentation by using absolute frequencies, consistent denominators, and simplified visual aids can promote better comprehension across literacy levels; and (4) structuring Behavioral Recommendations into concise, sequential action steps with links to relevant resources can reinforce practical applicability. These refinements would elevate both the scientific quality and communicative impact of diabetes health materials, thereby increasing the effectiveness of public health interventions.

### Limitations

This study has several limitations. First, the sample was restricted to provincial CDC websites and focused exclusively on diabetes-related materials, without including hospitals, nongovernmental organizations, or social media platforms, which are also important health communication channels. Future research should expand to a broader range of health topics and communication platforms to test the generalizability of the C-CCI. Second, all raters had medical or nursing backgrounds. While this ensured professional rigor, it may have introduced certain biases. Rater training yielded acceptable interrater consistency (*κ*=0.624), although potential bias from disciplinary homogeneity remains. Future studies could strengthen methodological reflexivity by adopting hybrid rating panels that combine professional experts with lay raters to systematically compare expert-oriented and user-centered assessments. In this approach, lay raters could be purposively sampled to reflect variation in health literacy, education, and regional backgrounds, enabling a more representative appraisal of communication quality under real-world conditions. Such iterative and diversified designs would not only enhance measurement robustness but also model continuous improvement in cross-rater reliability and interpretive balance.

### Conclusions

This study completed the cross-cultural translation and psychometric validation of the C-CCI, establishing a reliable and context-sensitive instrument for evaluating the clarity and actionability of health communication in Mainland China. Beyond measurement performance, the findings highlight the C-CCI’s potential as a standardized quality assurance tool to support systematic improvement in public health communication, in line with national efforts to advance health literacy and equitable access to health information [59].

By demonstrating stable performance across text, text-image, and video formats in diabetes-related materials from provincial CDC websites, the C-CCI shows strong potential for routine application in CDC practice in chronic disease communication. As exemplified in diabetes education, this framework can be extended to broader chronic disease portfolios and used to support longitudinal monitoring of health communication quality over time. Its integration into content development and review workflows could facilitate evidence-informed standardization of health materials nationwide, helping to reduce avoidable comprehension gaps and strengthen public engagement. Future research should further validate this approach across disease areas and communication contexts, consolidating the role of the C-CCI within China’s public health system.

## Abbreviations

AVE: average variance extracted
C-CCI: Chinese version of Clear Communication Index
CCI: Clear Communication Index
CDC: Centers for Disease Control and Prevention
CFA: confirmatory factor analysis
CFI: comparative fit index
CR: composite reliability
EFA: exploratory factor analysis
I-CVI: Item-level Content Validity Index
KMO: Kaiser–Meyer–Olkin
PEMAT: Patient Education Materials Assessment Tool
RMSEA: root mean square error of approximation
S-CVI/Ave: Scale-level Content Validity Index/Average
SAM: Suitability Assessment of Materials
